# Vernal keratoconjunctivitis in Down syndrome: a case report

**DOI:** 10.1186/s12886-023-02855-y

**Published:** 2023-03-17

**Authors:** Maria Cristina Artesani, Mariacristina Esposito, Diletta Valentini, Alberto Villani, Alessandro Giovanni Fiocchi, Luca Buzzonetti

**Affiliations:** 1 Allergy Unit, Bambino Gesù Children’s Hospital, IRCCS, Piazza Sant’Onofrio 4 00165 Rome, Italy; 2 Ophthalmology Unit, Bambino Gesù Children’s Hospital, IRCCS, Rome, Italy; 3 Pediatric Unit, Pediatric Emergency Department, Bambino Gesù Children’s Hospital, IRCCS, Rome, Italy; 4 Chair of Pediatrics, Department of Systems Medicine, University of Rome “Tor Vergata”, Rome, Italy

**Keywords:** Down syndrome, Vernal keratoconjunctivitis, Keratoconus

## Abstract

**Background:**

Down syndrome (DS) or Trisomy 21 is the most common chromosomal disease and is characterized by possible heart defects, cognitive impairment and visual disorders.

**Case presentation:**

We describe for the first time a 17-year-old Caucasian girl suffering from Down syndrome associated with vernal keratoconjunctivitis (VKC), a rare disorder of the anterior segment of the eye, characterized by intense photophobia, redness, watering eyes and itching due to an inflammatory-allergic reaction of the cornea and conjunctiva. On slit-lamp examination, the girl showed conjunctival hyperemia, papillary hypertrophy, giant papillae and corneal leukoma in right eye as a result of a previous corneal ulcer. A successful topical immunosuppressant therapy with cyclosporin 1% was started.

**Conclusion:**

So far, to our knowledge, this is the first description of VKC in a patient with DS. Finding an inflammatory-allergic disease such as VKC in DS is unusual but it must be taken into account because keratoconus, one of the most frequent eye pathologies in DS, can be secondary to an unrecognized VKC.

## Background

Down syndrome (DS) or Trisomy 21 is the most common chromosomal disease and is characterized by multiple malformations especially affecting the heart level, cognitive impairment and visual disorders. Recently, in a population of 1207 DS patients ophthalmological disorders were found up to 40.8%, mostly keratoconus (27.2%) and refractive error (35.9%) with the need for eyeglasses [1] but so far never cases of vernal keratoconjunctivitis (VKC).

VKC is a rare disorder of the anterior segment of the eye characterized by intense photophobia, redness, watering eyes and itching [2] due to an inflammatory-allergic reaction of the cornea and limbal, tarsal (or both) conjunctiva [3, 4].

We describe here one case of Down syndrome associated with VKC.

## Case presentation

**Fig. 1:**
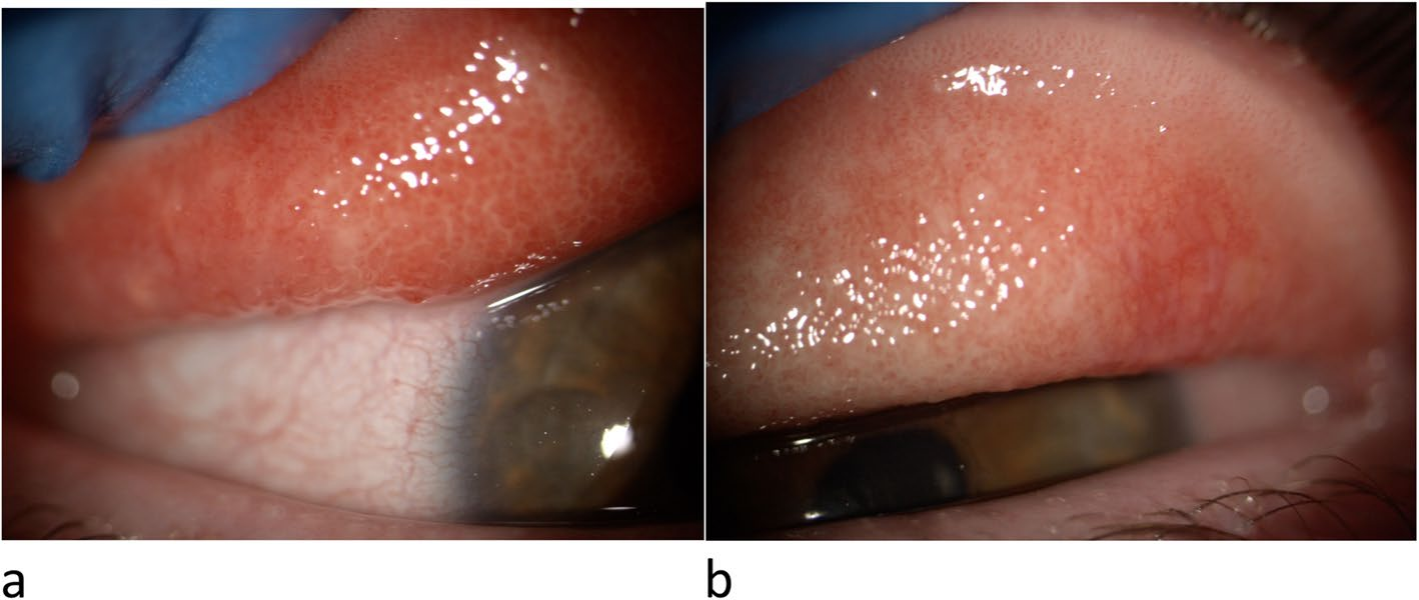
Slit-lamp examination, right (**a**) and left eye (**b**)

**Fig. 2:**
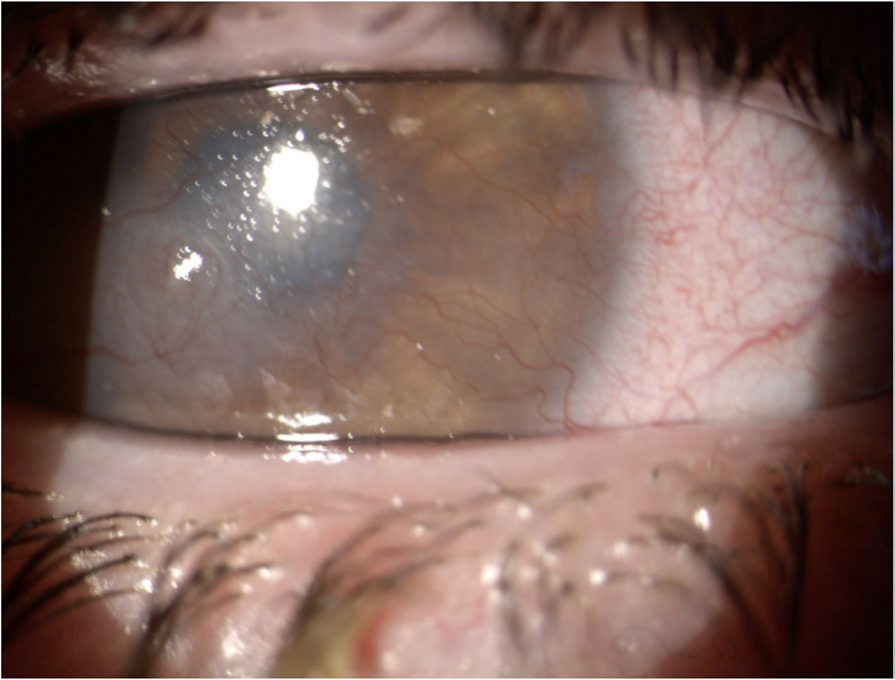
Right eye corneal leukoma

After obtaining informed consent and authorization of our Ethics Committee, we describe the case of a 17-year-old Caucasian girl suffering from Down syndrome who came to our observation due to the appearance of bilateral conjunctivitis. The patient complained of ocular itching with eye rubbing, burning, watering and mucoid stringy discharge and intense photophobia. Skin Prick Tests (Lofarma, Milan, Italy) for a standard panel of inhalant allergens were performed with negative result. On slit-lamp examination, the girl showed conjunctival hyperemia, papillary hypertrophy, giant papillae in both eyes (Fig. 1) and corneal leukoma in right eye (Fig. 2). VKC was diagnosed and the disease activity was graded, according to the Bonini VKC severity score [5], as moderate at time visit, although a complication of VKC is already present: in fact, the right eye corneal leukoma is the result of a previous corneal ulcer. A successful topical immunosuppressant therapy with cyclosporine 1% was started.

## Discussion and conclusions

To the best of our knowledge, at the time of writing, this is the first description of VKC in a patient with DS. In fact, in literature we have found no any other case of VKC as well as few studies on allergic disease in DS children, that probably indicates their poor likelihood of developing these diseases. Allergic sensitization is rare in DS individuals compared to the general population: they show low levels of specific IgE (7.6%) and fewer positive skin prick tests (18%) compared to non-DS children (40.2% and 54%, respectively) [6, 7]. Thus, finding an inflammatory-allergic disease such as VKC in DS is unusual but nevertheless possible as demonstrated by our case. Furthermore in patients affected by VKC itching induces eye rubbing with corneal epithelium microtrauma and damage. This, in susceptible individuals, can lead to cytokines release, myofibroblastst differentiation, biomechanical forces change and corneal tissue thinning with development of keratoconus [8, 9], a frequent complication of both VKC [10] and DS [1]. Therefore, our case suggests important implications for diagnostic workup and treatment. Before starting a specific treatment, in presence of suggestive symptoms, health professionals should consider other causes of specific symptoms associated with eye disease in DS, such as inflammatory-allergic ones. In this way, tailored therapies could be applied and corneal transplantation, required in 0.2% of DS with keratoconus, could be avoided [1, 11].

## Data Availability

The datasets used and/or analysed during the current study are available from the corresponding author on reasonable request.

## Abbreviations

DS: Down syndrome
VKC: Vernal keratoconjunctivitis
